# Metabolomics of oncogene-specific metabolic reprogramming during breast cancer

**DOI:** 10.1186/s40170-018-0175-6

**Published:** 2018-04-03

**Authors:** Chen Dai, Jennifer Arceo, James Arnold, Arun Sreekumar, Norman J. Dovichi, Jun Li, Laurie E. Littlepage

**Affiliations:** 1 Department of Chemistry and Biochemistry, University of Notre Dame, Notre Dame, IN 46556 USA; 2 Harper Cancer Research Institute, University of Notre Dame, 1234 N Notre Dame Avenue, South Bend, IN 46617 USA; 3 Department of Biochemistry and Molecular Biology, Baylor College of Medicine, Houston, TX 77030 USA; 4 Department of Applied and Computational Mathematics and Statistics, University of Notre Dame, Notre Dame, IN 46556 USA

**Keywords:** Breast cancer, Oncogene, Transgenic mouse model, Metabolic reprogramming, Metabolomics

## Abstract

**Background:**

The complex yet interrelated connections between cancer metabolism and oncogenic driver genes are relatively unexplored but have the potential to identify novel biomarkers and drug targets with prognostic and therapeutic value. The goal of this study was to identify global metabolic profiles of breast tumors isolated from multiple transgenic mouse models and to identify unique metabolic signatures driven by these oncogenes.

**Methods:**

Using mass spectrometry (GC-MS, LC-MS/MS, and capillary zone electrophoresis (CZE)-MS platforms), we quantified and compared the levels of 374 metabolites in breast tissue from normal and transgenic mouse breast cancer models overexpressing a panel of oncogenes (PyMT, PyMT-DB, Wnt1, Neu, and C3-TAg). We also compared the mouse metabolomics data to published human metabolomics data already linked to clinical data.

**Results:**

Through analysis of our metabolomics data, we identified metabolic differences between normal and tumor breast tissues as well as metabolic differences unique to each initiating oncogene. We also quantified the metabolic profiles of the mammary fat pad versus mammary epithelium by CZE-MS/MS. However, the differences between the tissues did not account for the majority of the metabolic differences between the normal mammary gland and breast tumor tissues. Therefore, the differences between the cohorts were unlikely due to cellular heterogeneity. Of the mouse models used in this study, C3-TAg was the only cohort with a tumor metabolic signature composed of ten metabolites that had significant prognostic value in breast cancer patients. Gene expression analysis identified candidate genes that may contribute to the metabolic reprogramming.

**Conclusions:**

This study identifies oncogene-induced metabolic reprogramming within mouse breast tumors and compares the results to that of human breast tumors, providing a unique look at the relationship between and clinical value of oncogene initiation and metabolism during breast cancer.

**Electronic supplementary material:**

The online version of this article (10.1186/s40170-018-0175-6) contains supplementary material, which is available to authorized users.

## Background

In the beginning of the 20th century, Otto Warburg and his colleagues described increased glycolysis in the presence of oxygen (aerobic glycolysis) in actively growing tumors [1]. Research has identified several critical metabolic pathways underlying cancer progression [2, 3]. However, after a century of extensive gene expression and protein profiling of human tumors, relatively little is known about the regulation of the metabolic changes that contribute to cancer development. Identifying these complex molecular events of cancer progression will identify potential biomarkers and therapeutic targets of cancer.

Oncometabolites are metabolites whose abnormal accumulation induces malignancy [1, 3–5]. An example of an oncometabolite is R-2-hydroxyglutarate (R2HG), which is produced by mutated isocitrate dehydrogenase [5–7]. Normally, isocitrate dehydrogenase (IDH1/2) converts isocitrate into α-ketoglutarate. However, when IDH1 is mutated in gliomas and acute myeloid leukemias (AML), R2HG is produced [7, 8]. The abnormal accumulation of R2HG causes global DNA hypermethylation by deactivating DNA demethylases, which causes upregulation of the WNT, NOTCH, and TGF-β pathways responsible for aberrant cell proliferation, differentiation, and cancer [6, 7, 9–11]. Two drugs AG-120 and AG-221 (Agios Pharmaceuticals, Cambridge, MA), which target the mutated IDH enzymes, are under phase 1 clinical trials (NCT02632708, NCT02677922). In addition to R2HG and the pathways regulated by it, other metabolic pathways may contribute to cancer and may define new cancer subtypes relevant to personalized treatment of cancer in patients.

While specific oncogene activation or tumor suppressor deactivation can reprogram the underlying metabolism of tumor tissue, few oncogenes and tumor suppressors have been investigated. A few of the genes studied as initiators of metabolic reprogramming include *MYC*, *KRAS*, and *BRCA1*. *MYC* promotes cell survival and proliferation by upregulating many crucial metabolic pathways, including glucose and glutamine catabolism as well as lipid synthesis and nucleotide synthesis [12, 13]. *MYC* also promotes the accumulation of oncometabolite R2HG in breast cancer [10]. *KRAS* enhances glycolysis but also reduces the oxidative flux through the TCA cycle and increases the utilization of glutamine as an energy source [14]. *BRCA1* can metabolically reprogram cells by decreased glycolysis, increased TCA cycle and oxidative phosphorylation, and decreased ketone bodies and free fatty acids [15, 16]. Despite all these studies, we still do not know the metabolic pathways that are reprogrammed by most oncogenes. In addition, many of the therapies used to treat patients are themselves regulated by metabolic pathways (e.g., processed/activated in the body, differentially utilized based on channel proteins or transporters), and metabolism can significantly impact treatment efficacy. Therefore, comparing the metabolic reprogramming induced by a panel of oncogenes will aid in the development of more individualized treatments that reverse the metabolic reprogramming.

Our study aims to identify the scope of the metabolic reprogramming that is caused by individual induced oncogenes by comparing the metabolic profiles of breast tumors induced by tumor-initiating oncogenes. Previous studies have identified genomic features of mouse breast cancer models and the relationship with human breast cancer [17–19]. In contrast to gene expression, the metabolomics features of mouse breast cancer models and the relationship with human breast cancer are not established. Both the tumor metabolic pathways that are differentially regulated within common transgenic mouse models and the comparison of mouse model data to human breast cancer clinical data are essential to increase our understanding of the relationship between initiating oncogenes, breast cancer phenotypes, and metabolism. Through detection of the metabolites that are significantly up/downregulated in different models of breast cancer, our ultimate goal is to identify key metabolites, metabolic pathways, or even novel oncometabolites that contribute to cancer progression or response to treatment.

## Methods

### Transgenic mouse models

Mice developed spontaneous tumors through the transgenic expression of the indicated oncogenes: MMTV-PyMT, MMTV-PyMT-DB, MMTV-Wnt1, MMTV-Her2/neu, and C3(1)-SV40 T-antigen (C3-TAg). All animal experiments were approved and conducted in accordance with the University of Notre Dame Institution Animal Care and Use Committee guidelines (protocol # 15-10-2724). Mice used in this study were maintained under pathogen-free conditions in the University of Notre Dame Freimann Life Sciences animal facility.

### Breast tumor sample preparation

Tumors were surgically removed and flash frozen after collection. Mammary gland tissues were collected and frozen from mice at ages ranging from 15 to 27 weeks of age for profiling. Frozen tissue samples were sent to Metabolon for metabolomics analysis. Samples were first removed of protein fraction and then divided for liquid chromatography (LC) and gas chromatography (GC) for compound separation and then scanned through mass spectrometry. The sample preparation process was carried out using the automated MicroLab STAR® system from Hamilton Company. Recovery standards were added prior to the first step in the extraction process for QC purposes. Sample preparation was conducted using a proprietary series of organic and aqueous extractions to remove the protein fraction while allowing maximum recovery of small molecules. The resulting extract was divided into two fractions: one for analysis by LC and one for analysis by GC. Samples were placed briefly on a TurboVap® (Zymark) to remove the organic solvent. Each sample was then frozen and dried under vacuum. Samples were then prepared for the appropriate instrument, either LC/MS or GC/MS.

### Liquid chromatography/mass spectrometry (LC/MS, LC/MS2)

This procedure was completed by Metabolon. The LC/MS portion of the platform was based on a Waters ACQUITY UPLC and a Thermo-Finnigan LTQ mass spectrometer, which consisted of an electrospray ionization (ESI) source and linear ion-trap (LIT) mass analyzer. The sample extract was split into two aliquots, dried, and then reconstituted in acidic or basic LC-compatible solvents, each of which contained 11 or more injection standards at fixed concentrations. One aliquot was analyzed using acidic positive ion-optimized conditions and the other using basic negative ion-optimized conditions in two independent injections using separate dedicated columns. Extracts reconstituted in acidic conditions were gradient eluted using water and methanol both containing 0.1% Formic acid, while the basic extracts, which also used water/methanol, contained 6.5 mM ammonium bicarbonate. The MS analysis alternated between MS and data-dependent MS2 scans using dynamic exclusion.

### Gas chromatography/mass spectrometry (GC/MS)

The samples destined for GC/MS analysis were re-dried under vacuum desiccation for a minimum of 24 h prior to being derivatized under dried nitrogen using bistrimethyl-silyl-triflouroacetamide (BSTFA). The GC column was 5% phenyl, and the temperature ramp is from 40 to 300 °C in a 16-min period. Samples were analyzed on a Thermo-Finnigan Trace DSQ fast-scanning single-quadrupole mass spectrometer using electron impact ionization. The instrument was tuned and calibrated for mass resolution and mass accuracy on a daily basis. The information output from the raw data files was automatically extracted as discussed below.

### Accurate mass determination and MS/MS fragmentation (LC/MS), (LC/MS/MS)

The LC/MS portion of the platform was based on a Waters ACQUITY UPLC and a Thermo-Finnigan LTQ-FT mass spectrometer, which had a linear ion-trap (LIT) front end and a Fourier-transform ion cyclotron resonance (FT-ICR) mass spectrometer backend. For ions with counts greater than 2 million, an accurate mass measurement could be determined. Accurate mass measurements could be made on the parent ion as well as fragments. The typical mass error was less than 5 ppm. Ions with less than two million counts required a greater amount of effort to characterize. Fragmentation spectra (MS/MS) were typically generated in data-dependent manner, but if necessary, targeted MS/MS could be used, such as in the case of lower level signals.

### Data extraction and quality assurance

The data extraction of the raw mass spec data files yielded information that was loaded into a relational database and manipulated without resorting to BLOB manipulation. Once in the database, the information was examined, and appropriate QC limits were imposed. Peaks were identified using Metabolon’s proprietary peak integration software, and component parts were stored in a separate and specifically designed complex data structure.

### Compound identification

Compounds were identified by comparison to library entries of purified standards or recurrent unknown entities. Known chemical entities were identified by comparison to metabolomic library entries of purified standards. As of this writing, more than 1000 commercially available purified standard compounds had been acquired registered into LIMS for distribution to both the LC and GC platforms for determination of their analytical characteristics. The combination of chromatographic properties and mass spectra gave an indication of a match to the specific compound or an isobaric entity. Due to the limitations of detection and database identification, not all detected metabolites are detected or named. Additional entities could be identified by their recurrent nature (both chromatographic and mass spectral). These compounds have the potential to be identified by future acquisition of a matching purified standard or by classical structural analysis.

### Normalization

**Fig. 1:**
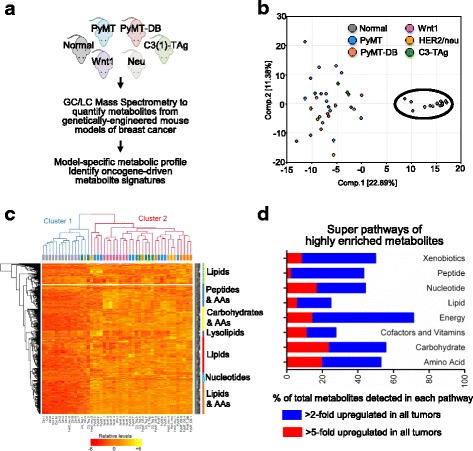
Overview of metabolomics data. **a** Experimental design. Breast tumors were collected from five transgenic mouse models and normal mammary tissue from littermates. Samples then were analyzed by GC-MS and LC-MS/MS to acquire metabolomics data, which was then used to acquire oncogene-specific metabolic profiles. **b** Principal component analysis (PCA) of tumor metabolites. Each group of tumor samples (multi-colored) are separated from normal mammary tissue samples (gray) and from other tumor models. **c** Unsupervised cluster analysis of 47 samples and 374 metabolites. Samples are separated by column, and metabolites are separated by row. All normal tissue was within cluster 1 (blue); tumors are nearly all in cluster 2 (red), generally with high metabolite levels. **d** Super pathways of universally enriched metabolites in all tumor groups compared to normal tissue. The *y*-axis indicates the metabolic super pathways of metabolites, and the *x*-axis indicates the percentage of metabolites in each super pathway. The bars indicate metabolites that were upregulated more than twofold (blue) or fivefold (red) in all five tumor groups compared to normal tissue. The number of metabolites belonging to each major pathway was normalized against the total number of metabolites detected in the same pathway. See also Additional files 1, 2, and 3.

For studies spanning multiple days, data were normalized to correct variation from instrument inter-day tuning differences. Each compound was corrected in run-day blocks by registering the medians to equal one (1.00) and by normalizing each data point proportionately (termed the “block correction”; Fig. 1). Studies that did not require more than one day of analysis did not require normalization except for purposes of data visualization.

### Separation of adipocyte-enriched and epithelial cell-enriched mouse mammary tissue

The breast tissues were harvested from three-week-old FVB mice prior to cutting and separating the epithelial enriched sample, which included the nipple, and the adipocyte enriched sample, which included no epithelium. A y-incision was made in the abdominal region of the mice to reveal the number 4 mammary gland. Two incisions were made to isolate the lymph node. The nipple side portion and the remaining portion of number 4 mammary gland were then collected in separate Eppendorf tubes. The samples were flash frozen in liquid nitrogen and kept frozen before CZE mass spectrometry analysis.

### Capillary zone electrophoresis mass spectrometry

#### Sample preparation

Fat pad and nipple tissues were homogenized using a CryoGrinder™ (OPS Diagnostics). Metabolites were extracted using cold acetonitrile-water (80:20 *v*/*v*). Five hundred microliter of extraction buffer was added to 20 mg of tissue, shaken vigorously for 2 min, and centrifuged (10,000 rpm × 5 min) [20]. The supernatant was collected, and the extraction procedure was repeated on the residue. The pooled supernatant was clarified by centrifugation at 13,000 rpm for 5 min. The clarified supernatant was dried in a vacuum concentrator and stored at − 20 °C until analysis.

#### CZE-ESI-MS conditions

Separations were completed using an uncoated 38 cm, 20-μm ID, 150-μm OD fused silica capillary. The distal tip of the capillary was etched to ~ 40 μM outer diameter with hydrofluoric acid [21, 22]. The metabolite extract (10 mg/mL) was hydrodynamically injected at 6 psi for 2 s, corresponding to a 26 nL volume. The separation buffer was 0.5% acetic acid, and the electrospray sheath liquid was composed of 0.5% formic acid, 10% methanol. The separation voltage was 16.6 kV, and the electrospray voltage was 1.2 kV. The capillary was coupled to an OrbiTrap Velos mass spectrometer (Thermo Fisher Scientific) using a third-generation electrokinetically pumped, sheath-flow interface [22]. Electrospray was generated through a borosilicate glass nanospray emitter pulled with a Sutter P-1000 micropipette puller with an exit orifice of 25 μm inner diameter.

#### Mass spectrometer operating parameters

Full MS scans were acquired in the Orbitrap mass analyzer over the m/z 100–500 range with a mass resolution of 70,000 (at m/z 200). The target value was 1.00E+06. The ion selection threshold was 2.50E+04 counts, and the maximum allowed ion accumulation times were 250 ms for full MS scans and 80 ms for tandem mass spectra. The dynamic exclusion time was set to 30 s.

#### Data analysis

Raw files were converted to mZXML using Proteowizard (http://proteowizard.sourceforge.net/). The converted files were submitted to XCMS Online metabolomics data processing platform. XCMS aligns data and automatically integrates and extracts peak intensities [23]. Feature selection produces a list of differentially expressed metabolites based on *P* value (*P* ≤ 0.05, ≥ 1.5 fold change). These species were matched against the LC-MS and GC-MS metabolite database and standards. CZE-MS unique features were identified using MS2 data, METLIN, Human Metabolome Database (HMD), LIPIDMAPS, and Mouse Multiple Tissue Metabolome Database (MMMDB).

### Statistical analysis

Statistical analyses were calculated with the program R (http://cran.r-project.org/). The metabolomics raw data were analyzed by significance tests and classification analysis, as indicated. For pair-wise comparisons, we used Welch’s *t* tests and/or Wilcoxon’s rank sum tests, as indicated. For statistical designs comparing more than two samples, we used ANOVA (e.g., repeated measures ANOVA).

### Gene expression analysis

Gene expression analysis was conducted with the Web MEV platform (http://mev.tm4.org). Gene normalization was done according to previously published protocol [17]. For fold change analysis comparing expression of different transgenic tumors versus other tumors, we used linear models for microarray data (LIMMA) [24].

Gene functional annotation clustering was completed with the DAVID bioinformatics tool [25, 26].

## Results

### Quantification of metabolites expressed in oncogene-driven mouse breast tumors

We hypothesized that individual oncogenes differentially regulate and reprogram the metabolism within breast tumors. Using GC/LC/CZE mass spectrometry, we quantified the global metabolite profiles of mouse tissues collected from normal wildtype mammary glands and breast tumors derived from a panel of transgenic mouse models (Fig. 1a). We quantified and clustered 374 named metabolites across 47 samples (12 normal and 35 tumor mouse mammary tissues generated from five transgenic mouse lines). The raw data and subsequent analyses are included in Additional file 1. The mouse lines examined were MMTV-PyMT [27], MMTV-PyMT-DB [28], MMTV-Wnt1 [29], MMTV-Her2/neu [30], and C3(1)-SV40 T-antigen (C3-TAg) (details in Additional file 2) [31]. The major metabolic pathways that are represented by the 374 metabolites are shown in Additional file 3.

Principal component analysis (PCA) of the metabolomics data identified variations across the samples based on their metabolic profiles and was used for quality control. Normal mammary tissue samples clustered together tightly (black circle), suggesting similar metabolic profiles, whereas the tumor samples for each oncogenic transgene were more heterogeneous and diverged from normal tissue (Fig. 1b). Component 1 separated normal mammary gland tissue samples from tumor samples, and adding component 2 separated PyMT tumors from the other tumor groups. Wnt1-induced tumors were the most heterogeneous and clustered away from the other tumor models.

### Global changes in tumor metabolic profiles independently distinguish breast cancer from normal mammary tissue

We next determined which metabolites changed globally across breast tumors compared to normal tissue. An average of 258 out of 374 metabolites (69%) varied significantly between tumors and normal mouse mammary tissue, with most of these metabolites upregulated in tumors (Additional file 4). Unsupervised cluster analysis of the metabolic data grouped the tumors predominantly into two major clusters, which distinguished normal from tumor tissues (Fig. 1c). Within the clusters, the metabolite levels primarily increased across tumor samples (cluster 2, right) compared to normal mammary tissue samples (cluster 1, left). The clustering of metabolites is listed by their metabolic pathways on the *y*-axis of the heatmap. In comparison to the globally upregulated amino acid and carbohydrate metabolites, the lipid metabolism of the tumor samples did not show such a singular trend. In addition to increased lipid metabolites, we also found groups of lipid metabolites with equal or lower levels in tumor samples compared to normal mammary tissue samples. These data suggest that metabolite signatures independently distinguish breast cancer tissue from normal mammary gland tissue.

We identified the major metabolic pathways that changed the most between normal and tumor tissue. We looked at the most highly enriched metabolites and grouped the metabolites with twofold (149 metabolites) enrichment in all tumor tissues compared to the normal tissue. Within these 149 metabolites, we further grouped the metabolites into the most highly enriched metabolites (fivefold enriched) (Fig. 1d, Additional files 5 and 6). The most highly enriched metabolites in tumors compared to normal tissue were carbohydrate and amino acid metabolites. Tumor tissues showed increased glucose metabolism, amino acid metabolism, and TCA cycle intermediates, all of which are consistent with increased energy production and anaplerotic contributions from amino acid catabolism (Additional file 1, pathway heatmap).

**Fig. 2:**
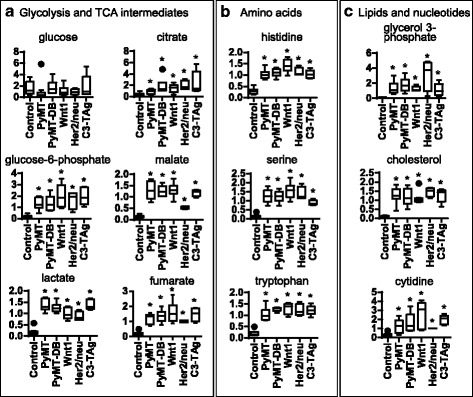
Metabolites supporting rapid growth are increased in tumors. Box and whisker plots of key metabolites of energy pathways and major catabolism/anabolism pathways in normal mammary tissue and tumors of transgenic mice. *y*-axis: scaled intensity of metabolites by mass spectrometry. Cross: mean value; center line: median; box lines: upper/lower quartile values; extended line: upper/lower extreme values; circles: outliers. **a** Glycolysis and TCA cycle intermediate levels are higher in all tumor groups compared to control, consistent with higher energy flux in rapid-growing tumors. **b** Amino acid metabolites are upregulated in all tumors compared to control. **c** Lipid and nucleotide pathway metabolites have higher levels in tumors, consistent with the need for more building blocks in rapid-growing tumors. *P < 0.05, Welch’s *t* test

Glucose metabolism provides energy and anabolic precursors and is increased in the tumors, as indicated by increased glycolytic and pentose phosphate intermediates, sorbitol and other hexoses, and glycogen metabolites (Fig. 2a, Additional file 1). Amino acid metabolism also increased, as indicated by elevated levels of free amino acids and their metabolites (Fig. 2b). The increased free amino acids may reflect not only increased catabolism but also increased amino acid uptake and/or protein degradation. Indeed, levels of dipeptides and modified amino acids (e.g., N6-acetyllysine) were higher in tumor than in normal tissue (Additional files 1 and 6). TCA cycle intermediates also increased and were consistent with increased energy production and anaplerotic contributions from amino acid catabolism (Fig. 2, Additional file 1). Finally, we also saw increased expression of phospholipid precursors, such as long-chain fatty acids and phospholipid turnover products in tumor tissues. Similarly, cholesterol uptake, marked by elevated cholesterol and the diet-derived sterol campesterol, and levels of nucleotide metabolites also increased in tumor tissues (Fig. 2c, Additional file 1). These changes were consistent with increased synthesis and remodeling of cellular membranes and increased nucleic acid production.

### Metabolic profiles of mammary gland fat pad and epithelium tissues suggest that the lipid differences seen in the breast tumors are unlikely to be caused by cellular heterogeneity

The differences between the metabolites of normal and tumor tissue might reflect actual differences in the metabolism of these tissues or alternatively might represent the cell heterogeneity, with varied metabolism by cell type, within these tissues. Normal mammary tissue is mostly composed of adipocytes, while breast tumors contain significantly more epithelium and less stroma [32, 33]. Thus, the lipid pools are expected to be quite different across normal and tumor tissue. Therefore, this cell heterogeneity between normal mammary and tumor tissue might sufficiently account for the metabolic differences between normal and tumor tissue. Differences could reflect cell heterogeneity rather than actual changes in the metabolism of the tissue.

**Fig. 3:**
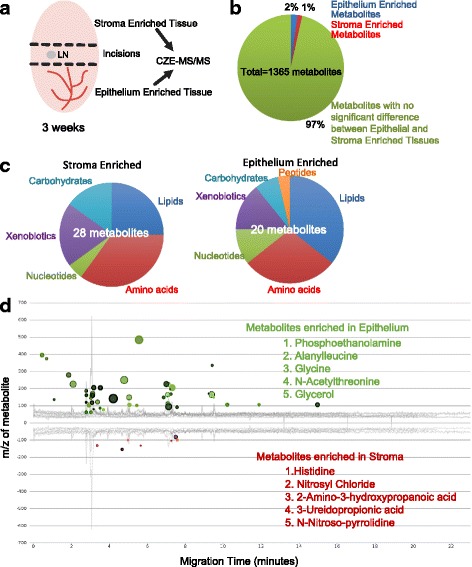
Metabolic differences between the epithelium enriched and the stroma enriched breast tissue. To determine if the metabolic differences we saw between normal mammary tissue and tumors are indeed due to cancer progression, rather than the difference in cell type heterogeneity, we compared the mammary gland metabolome of stroma enriched tissue (adipocyte rich) and epithelium enriched tissue during normal mammary gland development. **a** Overview of experimental design. Stroma enriched and epithelium enriched tissue were collected at 3 weeks, at the time when the mammary epithelium invades only a part of the mammary fat pad. **b** Of the 1365 different features detected, only 48 metabolites had significantly different levels. **c** Major pathway distribution of adipocyte enriched and epithelium enriched metabolites. **d** Cloud plot of all metabolites with significantly different levels in the two tissue groups. Each circle indicates one metabolite, with the color indicating the tissue detected in (green: epithelial cells, red: stroma). The size of the circles indicates the fold of change (compared to background noise), and the color of the circles indicate *P* values (darker color indicating lower *P* value). The most significantly changed metabolites (with the lowest *P* values) in both tissues were presented on the right side of the plot

We wanted to determine if the metabolic profile differences across our samples were due fundamentally to the differences in the types of cells available in equivalent masses of tissue, including changes in the fat pad stromal cell populations (e.g., adipocytes and lipid content in the mammary fat pad) of the tissue samples. To do this, we collected, quantified, and compared metabolites found within epithelium enriched and stromal enriched regions of a three-weekold mammary gland collected from a normal wildtype FVB/n mouse (Fig. 3). During mammary gland development, the epithelium invades from the nipple into the fat pad. Before puberty, the epithelium is restricted proximal to the nipple, while the majority of the fat pad contains no epithelium. We collected tissue samples at the start of puberty (3–4 weeks old) before the epithelium has invaded the whole fat pad and separated the proximal nipple end (epithelium enriched) from the distal fat pad (adipocyte enriched) (Fig. 3a). These samples were processed for CZE-MS/MS, and the epithelium enriched was compared to stroma enriched metabolic profiles (raw data in Additional file 7). The samples analyzed for metabolites included the regions proximal (epithelium enriched containing both epithelium and fat pad) and distal (stromal enriched containing fat pad devoid of epithelium) to the nipple. The metabolic differences between the epithelium enriched and stromal enriched tissue samples were then compared to the metabolites that differed between normal and cancer tissues to determine the metabolic contribution of stroma and epithelium of a normal mammary gland fat pad.

The results from our CZE-MS/MS metabolomics analysis identified 48 metabolites whose levels were significantly different between epithelium- and stromal-enriched tissues (Fig. 3b–d and Additional file 7). Of the 48 metabolites, 28 were higher in the epithelium enriched tissue, 20 were higher in the stroma enriched tissue (Fig. 3 and Additional file 7). Of the 28 epithelial enriched metabolites, most were amino acids, lipids, and nucleotide metabolites. Since these 48 metabolites represent less than 5% of the over 1300 metabolites detected in the CZE-MS/MS experiment (Fig. 3b, c), this suggests that most of the metabolite differences we observed between normal and tumor tissues are not due to cell heterogeneity but instead are due to metabolic changes of cancer progression. Therefore, the metabolic differences between the mammary tissue and cancer tissue could represent changes from within the cancer epithelial cells themselves or alternatively from stromal cells that are recruited to or modified by the normal fat pad stroma. However, based on these data, we cannot determine the contribution of proliferation to the metabolite differences between the normal mammary tissue and the tumor tissues.

### Metabolic profile of Wnt1 tumors suggests differences in eicosanoid, taurine, and one-carbon metabolism

We next compared the metabolites from each of the oncogene-induced tumor samples to identify metabolic profiles uniquely induced by each oncogene. We found metabolic profiles that significantly changed (increased/decreased) after expression of each oncogene compared to the other tumor and normal tissue (Additional files 1 and 8).

**Fig. 4:**
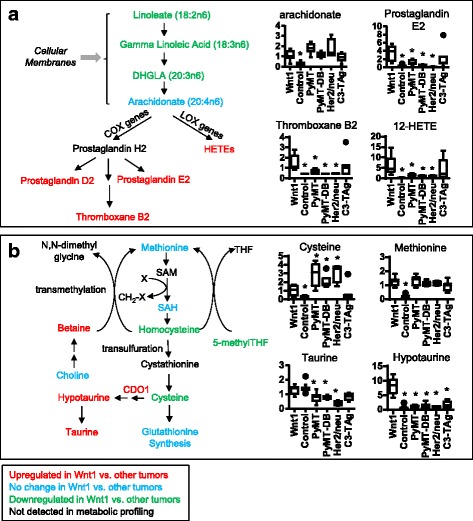
Metabolomic differences associated with Wnt1-initiated tumors. Metabolomics revealed significantly different eicosanoid and cysteine-methionine metabolites in Wnt1 tumors compared to other transgenic model tumors. **a** Eicosanoid metabolism. Left: the eicosanoid pathway, with colors indicating increased metabolite levels compared to at least three other tumor groups (red), decreased levels (green), no change (blue), or not detected (black). Right: Graphs of the quantification of eicosanoid precursors (AA) and some eicosanoids. Middle line of box plots indicates median of sample group. *P < 0.05, Welch’s *t* test. **b** Quantification of cysteine-methionine metabolism. CDO1 (black) converts cysteine to hypotaurine. *P < 0.05, Welch’s *t* test. Statistical comparisons were made between Wnt1 and every other sample group. Abbreviations: COX cyclooxygenase, LOX lysyl oxidase DHGLA dihomo-γ-linolenic acid, HETE hydroxyeicosatetraenoic acid, SAM *S*-adenosyl methionine, SAH *S*-adenosylhomocysteine, THF tetrahydrofolate, Cdo1 cysteine dioxygenase

The metabolic pathways of the Wnt1 tumors especially differed from the other tumor models. Wnt1 tumors contained significantly increased eicosanoids (e.g., prostaglandin E2, thromboxane B2, and 12/15-HETE), polyamines (putrescine, spermine, and spermidine), and taurine synthesis (decreased cysteine with increased hypotaurine and taurine) and decreased levels of free fatty acids and lysolipids (Fig. 4, Additional file 1). Also, a subset of metabolites from methionine and cysteine metabolism and one-carbon metabolism pathways were significantly differentially expressed in Wnt1 tumors compared to the other tumors. For example, Wnt1 tumors have *increased* taurine levels, while the other tumors *reduced* taurine levels compared to normal tissue.

One-carbon metabolism contributes one-carbon units in many biosynthetic metabolic pathways. One-carbon metabolism seems to be lower in Wnt1 compared to other tumors. Dipeptide levels were also higher in Wnt1 tumors than in the other tumors, indicating more active amino acid metabolism (Additional file 1). Levels of betaine (and isobars containing betaine-aldehyde and *N*-methyldiethanolamine), an amino acid synthesized de novo from choline, is also higher in the Wnt1 tumors than in the other tumor groups. Notably, the levels of 5-methyltetrahydrofolate (5-meTHF), the central metabolite of the one-carbon pathway, were lower in Wnt1 tumors compared to Her2/neu or PyMT tumors (Additional file 1). Consistently, the long-chain fatty acids and phospholipids decreased in Wnt1 compared to other tumors (Additional file 1, pathway heatmap).

### PyMT and PyMT-DB tumors differ in glucose, eicosanoid, and glycogen metabolism

**Fig. 5:**
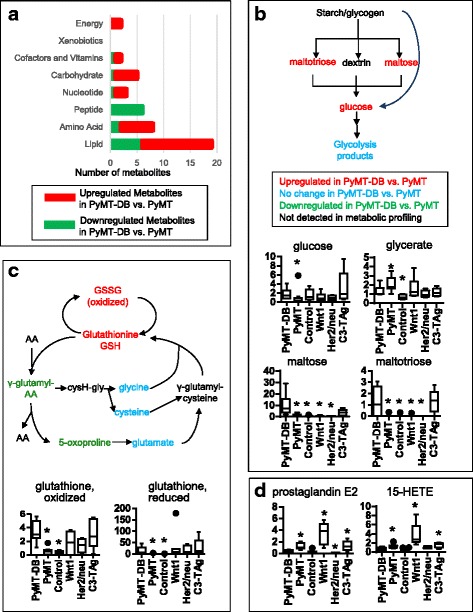
Metabolic comparison of PyMT vs. PyMT-DB tumors. PyMT-DB has increased glycolytic flux and more glycogen breakdown, as well as reduced inflammation, according to the metabolomics results. **a** Super pathway distributions of significantly up/downregulated metabolites in PyMT-DB tumors compared to PyMT tumors. Increases in energy, carbohydrate, amino acid, and lipid metabolism are apparent in PyMT-DB tumors, as is the decrease in peptide metabolites. **b** Starch/glycogen metabolism is significantly increased in PyMT-DB tumors compared to PyMT tumors. **c** Glutathione metabolism is altered in PyMT-DB compared to PyMT, with higher glutathione levels and lower γ-glutamyl cycle intermediates. **d** Eicosanoid levels are lower in PyMT-DB vs. PyMT. *P < 0.05, Welch’s *t* test. Statistical comparisons were made between PyMT-DB and every other sample group

Compared to the PyMT mouse, the PyMT-DB mouse contains two point mutations (Y315/322F) in the PyMT oncogene that prevent the activation of phosphatidylinositol 3-kinase [28]. The general metabolic differences between PyMT-DB and PyMT tumors included reduced glucose metabolism and eicosanoids and increased glycogen storage and glutathione in PyMT-DB tumors compared to PyMT tumors (Fig. 5a, Additional file 1 pathway heatmap). Glucose levels were higher in PyMT-DB tumors, while glycerate and lactate levels were lower (Fig. 5b). In addition, glycogen metabolites maltotriose and maltose were higher in PyMT-DB tumors, suggesting increased glycogen synthesis in PyMT tumors compared to PyMT-DB tumors (Fig. 5b). Eicosanoid and arachidonate levels also were lower in PyMT-DB tumors than in PyMT tumors (Fig. 5d, Additional file 1). Additionally, we observed increased levels of long-chain fatty acids in PyMT-DB compared to PyMT tumors, suggesting increased fat storage or decreased fat breakdown (Additional file 1).

Consistent with increased glutathione synthesis in these tumors, the levels of glutathione (both the reduced form GSH and the oxidized form GSSG) and ophthalmate, an analogous tripeptide that is also synthesized by the same enzymes as glutathione, were both higher in PyMT-DB tumors than in PyMT tumors (Fig. 5c). In addition, a number of γ-glutamyl amino acid levels (i.e., γ-glutamyl alanine, phenylalanine, and threonine) and 5-oxoproline levels decreased in PyMT-DB tumors relative to PyMT tumors (Fig. 5c, Additional file 1). The significantly lower 5-oxoproline levels and unchanged glutamate levels in PyMT-DB compared to PyMT tumors remain consistent with reduced turnover of glutathione via the γ-glutamyl cycle, possibly due to decreased γ-glutamyl transpeptidase activity.

### Metabolic profiles of HER2/neu tumors suggest altered lipid metabolism

**Fig. 6:**
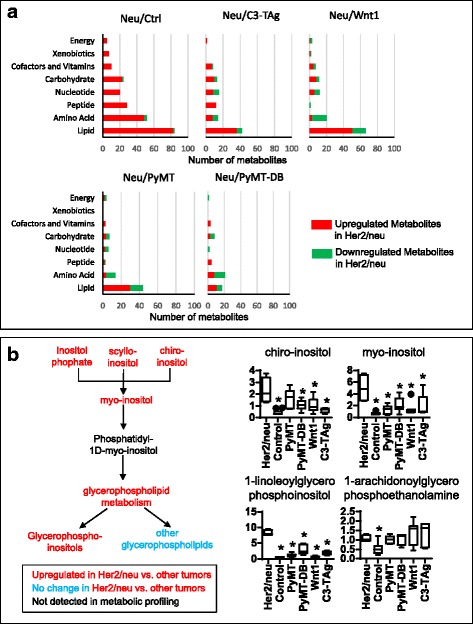
Metabolic profile of Her2/neu tumors. Her2/neu tumors have increased lipid metabolism compared to other tumors. **a** Super pathway distributions of significantly up/downregulated metabolites in Her2/neu tumors compared to tumors of other transgenic mouse models and normal mammary tissue. Elevated levels of lipid metabolites are observed in Her2/neu tumors compared to other tumors. **b** Inositol metabolites are upregulated in Her2/neu tumors compared to other tumors. *P < 0.05, Welch’s *t* test. Statistical comparisons were made between Her2/neu and every other sample group

The most prominent metabolic profiles of the Neu tumors suggested altered lipid metabolism. Neu tumors expressed higher levels of lipids, including long-chain fatty acids, polyunsaturated fatty acids, and inositol metabolism metabolites, compared to the other tumor cohorts (Fig. 6a, Additional file 1). The inositol pathway in particular stood out, since nearly all of the detected metabolites from this pathway were higher in Neu tumors than in other tumors (Fig. 6b, Additional file 1 pathway heatmap). Of the lysolipid metabolites examined, nearly all the phosphoinositols (e.g., 1-oleoylglycerophosphoinositol) were higher in Neu tumors as well. However, not all lipid metabolites are upregulated in Her2/neu tumors. Metabolites of acyl-carnitine metabolism were lower in Her2/neu tumors compared to the other models, with the exception of C3-TAg. Compared with other tumor models, altered lipid metabolism, followed by amino acids, best distinguished Her2/neu tumors from the other models. Metabolically, PyMT-DB is most similar to Her2/neu of the models examined.

### Metabolic profiles of C3-TAg tumors have decreased lipids and γ-glutamyl amino acids with increased glycogen metabolites

**Fig. 7:**
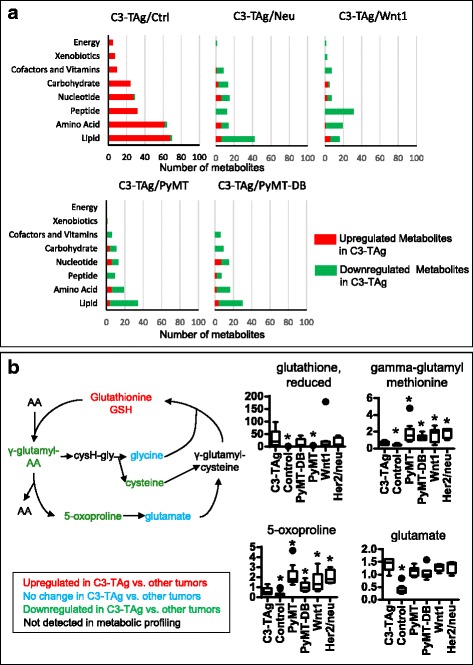
Metabolic profile of C3-TAg tumors. C3-TAg has lower metabolite levels in all pathways compared to other tumor groups, but still maintains higher levels of metabolites than normal mammary tissue. **a** Super pathway distributions of significantly up/downregulated metabolites in C3-TAg tumors compared to tumors of other transgenic mouse models and normal mammary tissue. **b** Decreased turnover of glutathione via the γ-glutamyl cycle in C3-TAg tumors compared to other tumors. *P < 0.05, Welch’s *t* test. Statistical comparisons were made between C3-TAg and every other sample group

In contrast to breast tumors from the other models, the metabolic profiles of C3-TAg tumors include pathways that overlap with Wnt1 tumors, including decreased free fatty acids and increased dipeptides, lysolipids, and eicosanoids (Fig. 7a, Additional file 1). In addition, of all of the tumor cohorts examined, C3-TAg tumors expressed the lowest levels of most types of metabolites (Fig. 7a). In particular, nearly all the γ-glutamyl amino acids are lower in C3-TAg than in other tumors, indicating either a rapid depletion of γ-glutamyl amino acids due to increased downstream demand or an upstream inhibition of γ-glutamyl amino acid production. In contrast, both the reduced (GSH) and the oxidized (GSSG) forms of glutathione are among the few metabolites that increased in C3-TAg tumors (Fig. 7b). Taken together, these data suggest an altered γ-glutamyl cycle in C3-TAg tumors, with either increased glutathione production or decreased glutathione breakdown. On the other hand, amino acid metabolite levels were similar between C3-TAg and the other tumors, except for cysteine, serine, and branched chain amino acids leucine and valine (Fig. 2b, Additional file 1).

Dipeptide levels and fat metabolism metabolites, especially phospholipid metabolites and polyunsaturated fatty acids, were generally lower in C3-TAg tumors than in other tumors (Additional file 1, pathway heatmap). Dipeptides are breakdown products of proteins and can also come from dietary sources. Several dipeptides have important functions, including as anti-oxidants or by stimulating proliferation [34, 35]. While glycolysis and TCA cycle intermediates did not change significantly, the C3-TAg tumors produced reduced levels of pentose metabolites, oligosaccharides, nicotinamide metabolites, and folate metabolites, compared to other tumors (Additional file 1).

Glycogen metabolite levels also greatly increased in C3-TAg tumors compared to other tumors, suggesting a more active glycogen metabolism in these tumors.

### Prediction of human patient survival with C3-TAg oncogene-specific metabolites

To determine if our metabolic profiles have clinical significance, we determined if the metabolites identified from our mouse study analysis had prognostic value in a human breast tumor cohort. The cohort contained 67 human breast tumors and 65 tumor-adjacent noncancerous tissues from an ethnically diverse group of patients [10]. The significance of the metabolites affecting survival was calculated by fitting a Cox proportional hazard model, using all of the survival times as the output and metabolites as the input. For the metabolites used as input, we used the list of model-specific metabolites identified from our metabolomics data (Additional file 8). In our analysis, we first tested each of the individual metabolites from the mouse model-specific metabolite list for their prognostic value. However, with the exception of citrate and nicotinamide adenine dinucleotide (NAD+), no individual metabolite was sufficient for prognostic value (Additional file 9).

We hypothesized that because of the complexity of the metabolic network, single metabolite cannot sufficiently capture all the changes in the network and, thus, would serve poorly as prognostic indicators. Alternatively, oncogene-induced metabolic signatures composed of a group of multiple  metabolites might have prognostic value in patients and serve as a biomarker of disease and disease progression.

We took the top ten metabolites with the smallest *p* values from each mouse model-specific metabolite signature and combined them into a single metabolic signature to predict patient survival (Table 1, Additional file 9). Surprisingly, of the different transgenic mouse models, only the C3-TAg metabolic signature composed of ten metabolites had statistical significance between patient outcome (*P* = 0.009). We next modified the number of metabolites that we included in the metabolic signatures. With fewer than ten metabolites per signature, the PyMT-specific metabolite signature also predicts patient survival with modest statistical significance (nine metabolites: C3-TAg, *P* = 0.03; PyMT, *P* = 0.04). Metabolic signatures of seven metabolites also produced modest prognostic value for C3-TAg (*P* = 0.02) and PyMT (*P* = 0.04) (Table 1, Additional file 9). This result confirms our hypothesis that a more complex metabolite signature is needed to predict patient survival. Additionally, the exact number of metabolites needed in such a prognostic signature may need to be determined by further statistical analysis.

**Table 1:** Survival prediction in human patients using model-specific metabolites

Value for met signature (no. of metabolites in signature)	*P* value (10)	*P* value (9)	*P* value (8)	*P* value (7)	Metabolites used in COX proportional hazard model
Normal	*0.002*	*0.001*	*0.003*	*0.01*	Citrate; nicotinamide adenine dinucleotide (NAD+); gamma-glutamyltyrosine; *S*-adenosylhomocysteine (SAH); glycerophosphorylcholine (GPC); gamma-glutamylglutamate; choline phosphate; ribose; mannitol^$^; Isobar: ribulose 5-phosphate^#^, xylulose 5-phosphate*
Wnt1	0.79	0.786	0.72	0.63	2-Linoleoylglycerophosphoethanolamine; glycylleucine; margarate (17:0); creatine; 10-nonadecenoate (19:1n9); threonate; cytidine 5′-diphosphocholine; eicosenoate (20:1n9 or 11)^$^; lysine^#^; 2-hydroxyglutarate*
Neu	0.82	0.746	0.65	0.55	2-Linoleoylglycerophosphoethanolamine; *N*-acetylaspartate (NAA); pipecolate; glycerol 3-phosphate (G3P); dihomo-linoleate (20:2n6); dihomo-linolenate (20:3n3 or n6); stearoyl sphingomyelin; N6-acetyllysine^$^; trans-4-hydroxyproline^#^; docosapentaenoate (n3 DPA; 22:5n3)*
C3-TAg	*0.009*	*0.03*	*0.02*	*0.02*	Gamma-glutamyltyrosine; choline phosphate; Isobar: ribulose 5-phosphate, xylulose 5-phosphate; ribitol; gamma-glutamylalanine; thymine; maltotriose; ribulose^$^; choline^#^; 5-oxoproline*
PyMT	0.05	*0.04*	0.07	*0.04*	Gamma-glutamyltyrosine; *S*-adenosylhomocysteine (SAH); choline phosphate; stearoylcarnitine; phenyllactate (PLA); Isobar: ribulose 5-phosphate, xylulose 5-phosphate; cystine; *N*-acetylaspartate (NAA)^$^; gamma-glutamylalanine^#^; 2-hydroxyglutarate*
PyMT-DB	0.61	0.57	0.48	0.37	Ribose; maltose; margarate (17:0); 10-nonadecenoate (19:1n9); maltotriose; cytidine 5′-diphosphocholine; urea; glutathione, oxidized (GSSG)^$^; oleate (18:1n9)^#^; 10-heptadecenoate (17:1n7)*

*P* values were calculated by fitting a Cox proportional hazard model using survival times as output and the indicated metabolites as input. *P* values that are < 0.05 are italicized

*The metabolite not included in the 9 metabolite signature. See also Additional file 9

^#^The metabolite not included in the 8 metabolite signature. See also Additional file 9

^$^The metabolite not included in the 7 metabolite signature. See also Additional file 9

Interestingly, while individual C3-TAg-specific metabolites did not have prognostic value, the combined C3-TAg-specific metabolites became the best signature of prognostic outcome. This increase in predictive power demonstrates the need for a complex metabolic signature in studying such a complicated system as cancer metabolism.

Upon a closer inspection of C3-TAg-specific metabolites that were good prognostic markers, we noticed many metabolites in the γ-glutamyl cycle, as well as metabolites of the glycogen pathway. These two pathways are among the most prominent differences in our metabolomics data that separate C3-TAg tumors from other tumors.

### C3-TAg alters gene expression and metabolism of breast tumors

After identifying C3-TAg-specific metabolites as the best prognostic indicators of human patient survival among the different transgenic mouse models, we further investigated the similarities in metabolites and gene expression between the C3-TAg and human tumor models. We determined if changes in gene expression in the identified metabolic pathways could account for some of the metabolite differences. To do this, we compared our metabolomics data to a previously published gene expression dataset that compares the gene expression patterns of genetically engineered mouse models and human breast cancer patients [17]. The study included samples from numerous mouse models, including most of the ones used in our study as well as some that express the TAg oncogene under different promoters. Since their analysis did not distinguish between C3-TAg-induced gene expression in tumors, we also conducted our own analysis of the data using C3-TAg as a separate cohort (Additional file 10). In our analysis, we also limited the comparisons by including only data from the mouse models used in our metabolomics study (C3-TAg, Neu, PyMT, Wnt1).

The number of genes with expression level changes that either increased more than double or decreased more than half in C3-TAg breast tumors, compared to all other tumors, was larger than the number of genes changed in the other breast tumors (564 genes for C3-TAg, 61 for Neu, 41 for PyMT, 46 for Wnt1) (*P* < 0.05, LIMMA). This suggests that C3-TAg has the most distinct global gene expression profile of all of the mouse models analyzed.

The significant variations in gene-specific expression in C3-TAg tumors are consistent with the significant metabolic differences that we observed in the metabolic profile of C3-TAg tumors and may account for some of the observed metabolic changes. For example, our metabolomics study discovered that C3-TAg tumors had both globally reduced metabolite levels and reduced expression levels of metabolism-related genes, including glutathione-related genes, compared to the other mouse models. For example, the C3-TAg tumors had reduced expression of several genes responsible for GSH breakdown compared to other tumors. These genes included γ-glutamyl transferase (Ggt1) and glutathione S-transferase (Gstt2 and Gstt3). Taken together, this suggests that the change in γ-glutamyl cycle is due to a decreased breakdown of GSH that produces γ-glutamyl amino acids, which are part of the prognostic indicators of patient survival.

C3-TAg tumors also had increased expression of genes related to proliferation and division as well as nucleotide synthesis. Taken together with the metabolomics data, this suggests that the C3-TAg tumor cells undergo rapid cell division while at the same time reduce many of the biosynthetic metabolism pathways.

The increased expression of glycogen metabolism genes in C3-TAg tumors remains consistent with the gene expression data. This includes increased expression of the Phk1 (phosphorylase kinase regulatory subunit alpha 1) gene, which encodes for the alpha subunit of phosphorylase b kinase enzyme for activating phosphorylase b to increase glycogen breakdown. Ppp1r2 and Ppp1r3c expression also increased, leading to inhibition of protein phosphatase 1 (Pp1), which in turn keeps glycogen synthase in the inactive form.

Taken together, results from gene expression analysis are largely consistent with our metabolomics data. Combining metabolomics data with gene expression data allowed us to look at whole cellular processes in a way that is not possible using either single dataset and helps to identify better candidates for key regulators of metabolism.

## Discussion

In this study, we hypothesized that individual oncogenes regulate multiple metabolic pathways within tumor tissue, much like oncogenes regulate gene expression. We investigated the downstream metabolic changes driven by specific oncogenes during breast cancer by quantifying the metabolites of transgenic mouse breast tumor tissue. Using breast tumors from a panel of oncogene-driven transgenic mice, we identified the metabolite signatures induced by specific oncogenes during breast cancer metabolism. From the metabolic profiling, we identified both metabolic profiles that differed between normal and tumor tissues as well as metabolic profiles specific to each particular oncogene-driven breast tumor model. Across tumor models we see global increases in metabolites across many metabolic pathways, including those consistent with the metabolism of rapidly proliferating cells. These results suggest that individual oncogenes are able to induce metabolic reprogramming of the tumor tissue. Based on our analysis that compared the metabolites of the mammary gland epithelium and stroma enriched samples, the observed metabolic changes are unlikely due to differences in the cell populations represented in tumor tissue and are most likely result from changes induced by oncogene initiation.

### Oncogene-specific metabolic reprogramming during breast cancer

Some of the oncogene-specific metabolic profiles are consistent with known phenotypes and signaling pathways of the specific oncogenes. Alternatively, other metabolic profiles are unique to this dataset and might point us towards new directions to study how these mouse models mimic human disease and aid in selecting a personalized therapy for the tumor.

In particular, Her2/neu tumors had the highest and C3-TAg the lowest number of increased metabolites compared to other tumors, while the Wnt1 tumors were the most heterogeneous and produced the most significantly different metabolites compared to other models. The PyMT and PyMT-DB tumors were most similar, followed by PyMT-DB and Her2/neu tumors.

Multiple metabolic pathways were significantly different in Wnt1 tumors compared to other models, including a major shift in nutrient metabolism centered around the one-carbon metabolism. One-carbon metabolism serves as an integrator of nutrients, incorporating nutrients from various sources, such as amino acids from diet and de novo synthesis, and regulating the output in nucleotide and phospholipid synthesis to impact many aspects of nucleotide, amino acid metabolism, and lipid metabolism, including lipid storage [36]. Compared to other tumor models, Wnt1 tumors showed a significant increase in metabolites feeding into the one-carbon metabolism, dipeptide levels, and de novo synthesized amino acids, indicating more active protein metabolism. On the other hand, metabolites in the one-carbon metabolism (such as 5-meTHF) and in the downstream metabolic pathways regulated by one-carbon metabolism (such as lipid and nucleotide synthesis) are lowered in Wnt1 tumors compared to other tumors. Taken together, this seems to indicate a decrease in nutrient utilization through the one-carbon metabolism pathway. Also, Wnt1 tumors accumulated hypotaurine and taurine, which would have relied on the activation of tumor suppressor cysteine dioxygenase 1 (Cdo1) [37, 38]. Finally, we saw a marked increase in eicosanoid levels in Wnt1 tumors compared to other tumors, suggesting increased inflammation.

In comparing the metabolites expressed by the MMTV-PyMT and MMTV-PyMT-DB tumors, our data support a role for the PI3K/AKT pathway in regulating glucose uptake, glycogen metabolism, fat storage, and inflammation (Fig. 5, Additional files 1 and 4). These models express the same driver oncogene polyomavirus middle T antigen (PyMT), which leads to downstream activation of MAPK and PI3K/Akt [39]. However, the PyMT-DB mouse contains two point mutations (Y315/322F) in the PyMT oncogene that prevent the activation of phosphatidylinositol 3-kinase [28]. Therefore, the metabolic differences observed between the PyMT and PyMT-DB tumors should reflect the differential activation of the PI3K-AKT pathway between the two models. Consistent with our data, Her2/neu and PyMT-DB shared the most similar metabolic profiles (Fig. 6a), since they both predominantly activate the MAPK pathway [39, 40]. Our data showed decreased glycogen synthesis and decreased glucose uptake as a result of PI3K/AKT deactivation in the PyMT-DB tumors compared to PyMT tumors. In general, in PyMT-DB, levels increased of glucose, glycerides, glycogen metabolites, and some TCA intermediates, while levels of glycolysis intermediates decreased compared to PyMT. Consistent with our findings, Akt activation increases glycolysis [41, 42]. AKT activation also increases citrate and TCA cycle activation by the action of ATP citrate lyase (ACL), which is important for de novo lipid synthesis from citrate [41, 43]. The increased citrate levels that we observe in PyMT-DB compared to PyMT may, thus, reflect decreased activation of ACL. Our data also support roles for the PI3K/AKT pathway in fat storage and inflammation. The increased levels of long-chain fatty acids in PyMT-DB suggest increased fat storage or decreased fat breakdown in these tumors compared to PyMT tumors. On the other hand, the eicosanoid levels of PyMT-DB tumors are significantly lower than those of PyMT tumors, a sign of less inflammation in the former [44]. Finally, glutathione levels increased in PyMT-DB tumors compared to PyMT. These data are consistent with the observation that AKT activation increases reactive oxygen species (ROS) accumulation and inhibits ROS scavengers [42].

Of the tumor models analyzed, Neu tumors made the most lipid metabolites, which include storage fatty acids and signaling-related phospholipids. These metabolites included increases in long-chain fatty acids, polyunsaturated fatty acids, phospholipids, and inositols. Interestingly, we also saw a decrease in acyl-carnitine fatty acid metabolism, which is responsible for converting fatty acids into energy. This reduction of acyl-carnitine fatty acid metabolism may indicate reduced consumption of fatty acids and corresponds to the accumulation of long-chain fatty acids in Her2/neu tumors. Reduced eicosanoids alongside increased eicosanoid precursors, such as lineolate and dihomo-linolenate, suggest inhibition of eicosanoid production.

Finally, C3-TAg tumors showed the most distinct metabolic reprogramming of the tumor models that were compared to other tumors. The γ-glutamyl cycle is involved in many important functions such as the transport of amino acids across membranes and the synthesis and degradation of glutathione [45, 46]. Nearly all of the γ-glutamyl amino acids detected in our study were lower in C3-TAg tumors compared to other tumors, taken together with lowered 5-oxoproline levels and slightly increased glutathione levels, indicate a lowered turnover of glutathione via the γ-glutamyl cycle. Reduced levels of lipid metabolites, including polyunsaturated fatty acids, phospholipids, and lysolipids), suggests either increased lipid usage or decreased synthesis/storage in the C3-TAg tumors. C3-TAg tumors also have lower levels of nucleotide metabolites, especially nicotinamide metabolism. Interestingly, despite having low levels in many carbohydrate metabolites such as galactose, lactose, and some pentose metabolites, C3-TAg tumors have comparable levels of glycolysis and TCA intermediates. Taken together, C3-TAg tumors have the least accumulation of all major types of metabolites but still maintained energy metabolism with a rate similar to the other tumor models. This may indicate faster proliferation where cells are constantly dividing and using up all nutrients, or a more quiescent metabolic type with only the energy pathway on full load.

The metabolic reprogramming of tumors undoubtedly influences the tumors’ abilities to metabolize therapies and to develop resistance to treatments. As such, understanding the global metabolic reprogramming induced by expression of mutated oncogenes within tumor tissue is a novel strategy that ultimately identifies candidate treatments with efficacy against therapy-resistant cancers [47]. Global metabolic profiles resulting from even a well-studied pathway such as the PI3K/Akt pathway can aid in identifying additional tumor metabolic pathways influenced by PI3K/Akt signaling. For example, expression of oncogenic Akt in human mammary epithelial cells causes the metabolic reprogramming of multiple metabolic pathways that include the regulation of glycolysis and glutathione biosynthesis [48]. Increased glutathione biosynthesis was sufficient to cause resistance to therapy, while inhibition of glutathione biosynthesis was efficacious at treating the tumor as a combination therapy with an Akt pathway inhibitor. These data suggest that targeting a tumor’s addiction to particular metabolic pathways, such as glutathione biosynthesis, could be a new Achilles’s heel used in treating previously unresponsive tumors.

## Conclusion

Our understanding of the global metabolic reprogramming induced by oncogenes or loss of tumor suppressors is limited to date [47]. In summary, in this study, we identified unique metabolic profiles in breast tumors that are induced by a panel of oncogenes. By comparing the metabolic profiles of tumor models induced by specific oncogenes, we were able to identify metabolic pathways that are differentially regulated by each oncogene. By comparing these profiles to human metabolomics data, we uniquely identified C3-TAg tumor models as having prognostic value and being similar to the metabolomics of human breast tumors. This study identifies unique pathways that are candidate therapeutic targets for the treatment of breast cancer for the indicated tumor models. Future research will be required to look at the tumor metabolic programs induced by both genomic events as well as in response to treatment.

## Additional files

Additional file 1: Raw data for GC/LC-MS/MS metabolomics. Related to Figs. 1 and 2. This table contains all the GC/LC-MS/MS metabolomics data. Included under separate tabs are raw data, statistical analysis, pathway analysis, heat maps, and box plots. (XLSX 2322 kb)

Additional file 2: Mouse models used in the study. Related to Fig. 1. This table includes the mouse models and the number of mice in each group, including mouse genotype, age, and weight of sample sent. (XLSX 13 kb)

Additional file 3: Super pathways of all detected metabolites. Pie graphs show the super pathway distribution of all metabolites detected. It is used as a background for Fig. 1d. (PPTX 73 kb)

Additional file 4: Statistical comparison between sample groups. Related to Fig. 1. Welch’s two-sample t-test was used to identify metabolites significantly different between two sample groups. Columns indicate the groups compared, while rows indicate the number of up/downregulated (red/green) metabolites. The first row with numbers summarizes the number of metabolites that achieved statistical significance (*p* ≤ 0.05), while the second and third row contain breakdowns of up/downregulated metabolites. The next rows contain the number of metabolites approaching significance (0.05 < *p* < 0.10). The differences between normal tissue and tumors are much larger (> 200 significantly different metabolites) than the differences among different tumor groups themselves (~ 100 significantly different metabolites). This shows that while cancer and normal significantly differ in metabolic profiles, there is significant heterogeneity among tumor models as well. (DOCX 14 kb)

Additional file 5: List of greatly upregulated metabolites in tumors. Related to Figs. 1 and 2. Metabolites that are significantly upregulated (*p* < 0.05, Welch’s t-test) in all 5 tumor groups compared to normal are then screened for mean values. (A) Metabolites that are two-fold upregulated in all 5 tumor groups. (B) Metabolites that are five-fold upregulated in all 5 tumor groups. (XLSX 15 kb)

Additional file 6: Sub-pathway distribution of universally upregulated metabolites in all tumor groups. Supplementing Fig. 1d, this figure shows the sub metabolic pathways of universally upregulated metabolites in all tumor groups compared to normal mammary tissue. X-axis indicates percentage of all metabolites detected in each sub pathway that is upregulated, while y-axis indicates the sub pathways. (PPTX 361 kb)

Additional file 7: Raw data for CZE-MS/MS and list of significantly different metabolites from epithelial cells to adipocytes. Related to Fig. 1. This table contains all the metabolomics information for the fat pad/epithelium experiment, including raw data, statistical analysis, and identities of the significantly different metabolites. (XLSX 694 kb)

Additional file 8: List of model specific metabolites. Related to Fig. 2. Model specific metabolites were determined by non-parametric t-test comparing one mouse model to all other groups. The metabolites listed have a *p*-value smaller than 0.05. (XLSX 15 kb)

Additional file 9: Raw Data for Patient Survival Prediction Using Model Specific Metabolites. Related to Table 1. Using a Cox proportional hazards model, different metabolites and sets of metabolites were used as input to predict patient survival in a previously published human breast cancer patient metabolomics dataset. The *p*-values for each individual metabolite as well as a combination of the metabolites are listed. (XLSX 11 kb)

Additional file 10: Gene expression analysis of transgenic mouse models. Using previously published gene expression data from breast tumors of transgenic mouse models [17], we found genes with significantly different expression in each transgenic model compared to all other transgenic models used in our study. For a complete analysis using multiple other transgenic mouse model gene expression as backgrounds (including the ones we used), please refer to the supplementary files of the cited research article. (ZIP 463 kb)

## Abbreviations

5-meTHF: 5-Methyltetrahydrofolate
ACL: ATP citrate lyase
Akt: Protein kinase B
AML: Acute myeloid leukemia
ANOVA: Analysis of variance
BRCA1: Breast cancer 1
C3-TAg: C3(1)-Simian vacuolating virus 40 T-antigen
CDO1: Cysteine dioxygenase 1
COX: Cyclooxygenase
CZE: Capillary zone electrophoresis
DHGLA: Dihomo-γ-linolenic acid
ESI: Electrospray ionization
FT-ICR: Fourier-transform ion cyclotron resonance
FVB: FVB Friend virus B-type (mouse)
GC-MS: Gas chromatography mass spectrometry
GSH: Reduced glutathione
GSSG: Oxidized glutathione
Her2/neu: Human epidermal growth factor receptor 2
HETE: Hydroxyeicosatetraenoic acid
IDH: Isocitrate dehydrogenase
KRAS: V-Ki-ras2 Kirsten rat sarcoma viral oncogene homolog
LC-MS/MS: Liquid chromatography tandem mass spectrometry
LIT: Linear ion-trap
LOX: Lysyl oxidase
MAPK: Mitogen-activated protein kinase
MMTV: Mouse mammary tumor virus
PCA: Principal component analysis
PI3K: Phosphatidylinositol-4,5-bisphosphate 3-kinase
PyMT: Polyomavirus middle T antigen
R2HG: R-2-hydroxyglutarate
ROS: Reactive oxygen species
SAH: *S*-Adenosylhomocysteine
SAM: *S*-Adenosyl methionine
TCA cycle: Tricarboxylic acid cycle
TGF-β: Transforming growth factor beta
WNT: Wingless/integrated
