# Supplementary figures and images for: Metabolomics of oncogene-specific metabolic reprogramming during breast cancer
Source: Cancer Metab. 2018 Apr 3;6:5. doi: 10.1186/s40170-018-0175-6 (PMC5881178; doi:10.1186/s40170-018-0175-6)

## Slide 1
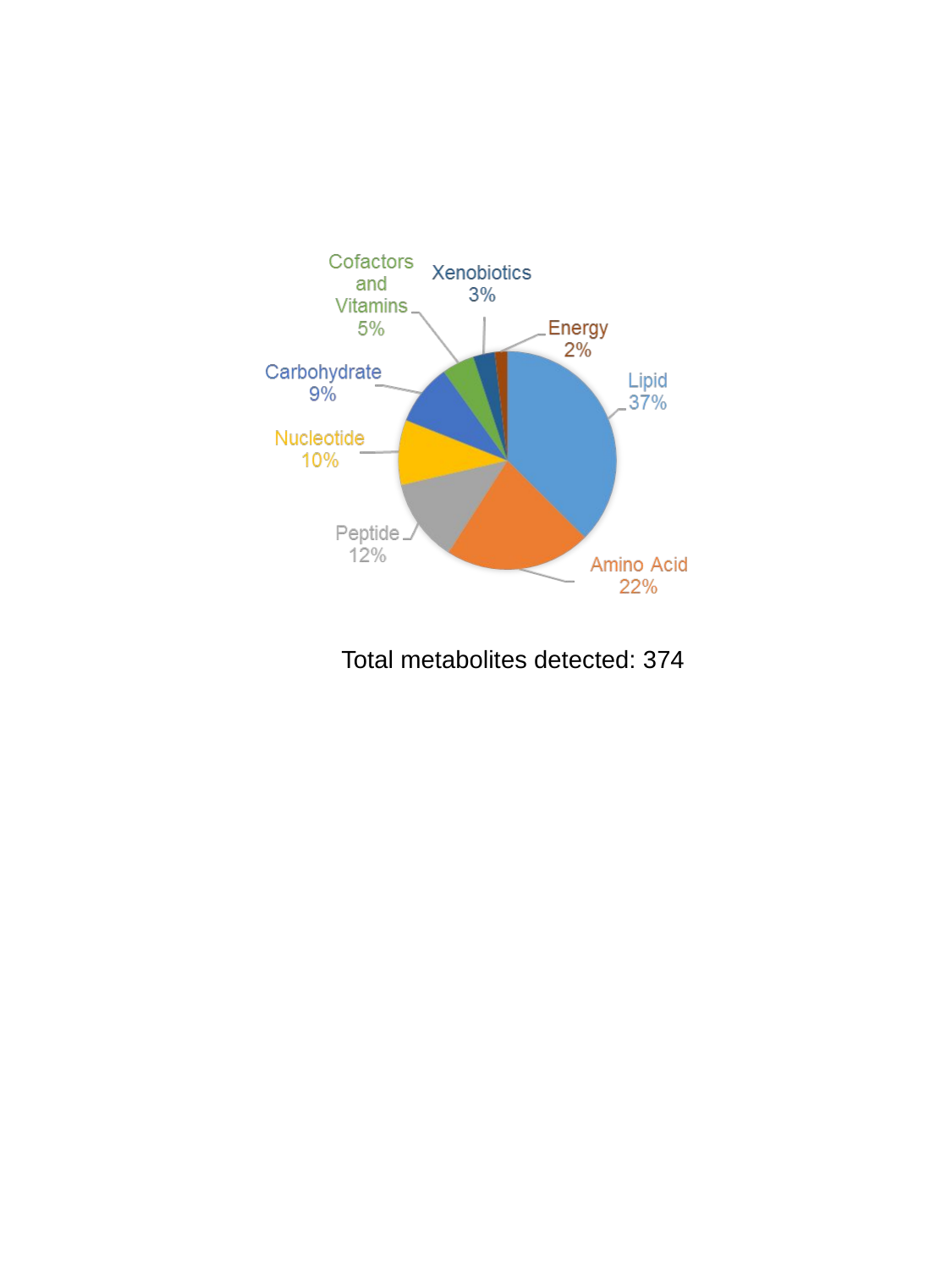

Total metabolites detected: 374

Supplement: Supplementary file 3 — Super pathways of all detected metabolites. Pie graphs show the super pathway distribution of all metabolites detected. It is used as a background for Fig. 1d. (PPTX 73 kb) [file 40170_2018_175_MOESM3_ESM.pptx]

## Slide 1
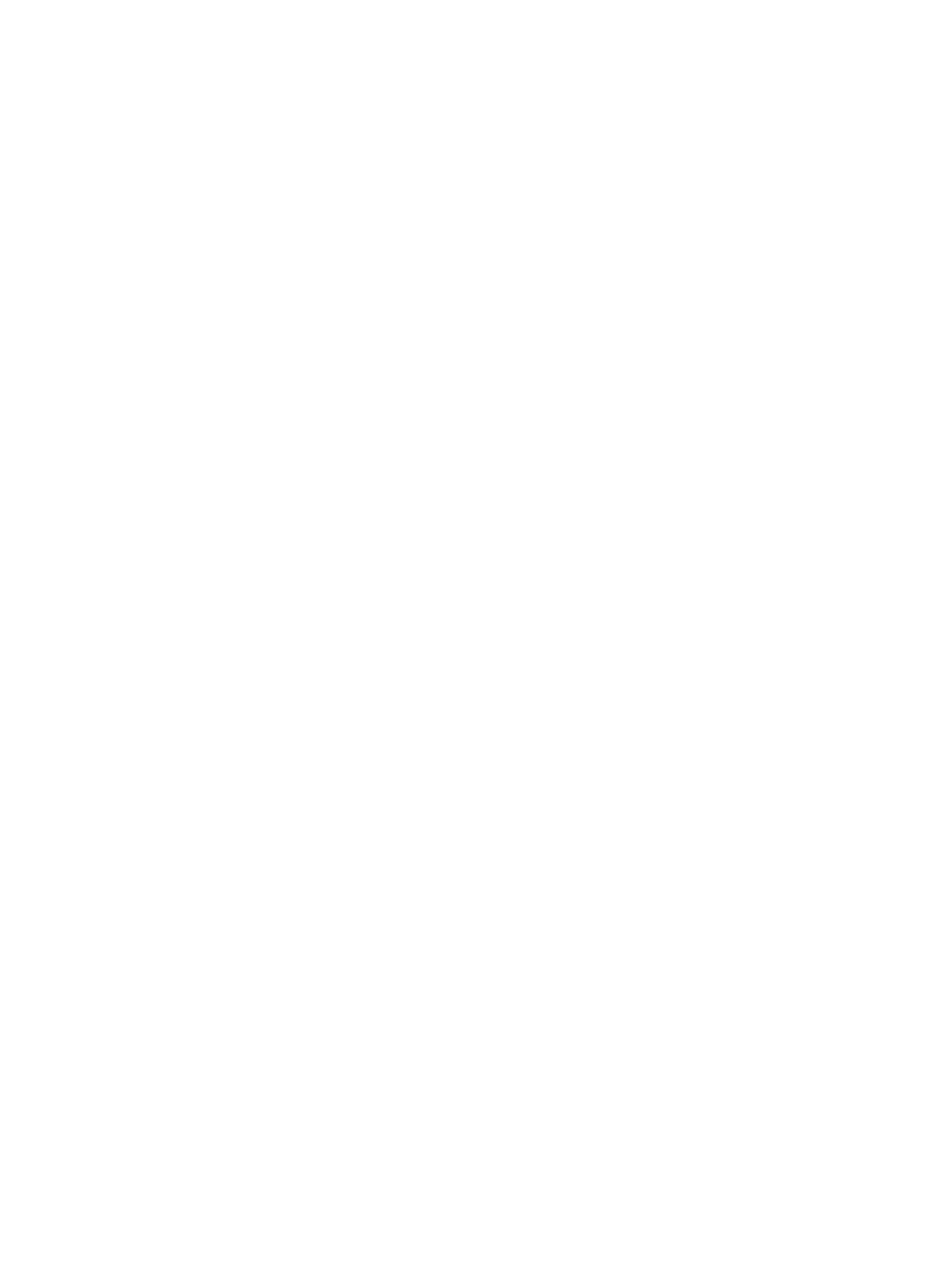

Supplement: Supplementary file 6 — Sub-pathway distribution of universally upregulated metabolites in all tumor groups. Supplementing Fig. 1d, this figure shows the sub metabolic pathways of universally upregulated metabolites in all tumor groups compared to normal mammary tissue. X-axis indicates percentage of all metabolites detected in each sub pathway that is upregulated, while y-axis indicates the sub pathways. (PPTX 361 kb) [file 40170_2018_175_MOESM6_ESM.pptx]
