# Supplementary material for: Metabolomics of oncogene-specific metabolic reprogramming during breast cancer
Source: Cancer Metab. 2018 Apr 3;6:5. doi: 10.1186/s40170-018-0175-6 (PMC5881178; doi:10.1186/s40170-018-0175-6)
Supplement: Supplementary file 4 — Statistical comparison between sample groups. Related to Fig. 1. Welch’s two-sample t-test was used to identify metabolites significantly different between two sample groups. Columns indicate the groups compared, while rows indicate the number of up/downregulated (red/green) metabolites. The first row with numbers summarizes the number of metabolites that achieved statistical significance (p ≤ 0.05), while the second and third row contain breakdowns of up/downregulated metabolites. The next rows contain the number of metabolites approaching significance (0.05 < p < 0.10). The differences between normal tissue and tumors are much larger (> 200 significantly different metabolites) than the differences among different tumor groups themselves (~ 100 significantly different metabolites). This shows that while cancer and normal significantly differ in metabolic profiles, there is significant heterogeneity among tumor models as well. (DOCX 14 kb) [file 40170_2018_175_MOESM4_ESM.docx]

| **Additional File 4. Statistical Comparisons among Tumor Models Related to Figure 1 and Figure 2** | | | | | | | | | | | | | | |
| --- | --- | --- | --- | --- | --- | --- | --- | --- | --- | --- | --- | --- | --- | --- |
| **Welch’s Two-Sample *t*-Test** | **PyMT vs. Ctrl** | **PyMT-DB vs. Ctrl** | | **Wnt1 vs. Ctrl** | | **C3-Tag vs. Ctrl** | | **Her2/neu vs. Ctrl** | | **Her2/neu vs. PyMT** | | **Her2/neu vs. PyMT-DB** | | **Her2/neu vs. C3-TAg** |
| **Total biochemical *p*≤0.05** | 277 | 268 | | 277 | | 240 | | 230 | | 81 | | 56 | | 105 |
| **Upregulated biochemicals** | 268 | 264 | | 259 | | 235 | | 225 | | 48 | | 29 | | 83 |
| **Downregulated biochemicals** | 9 | 4 | | 18 | | 5 | | 5 | | 33 | | 27 | | 22 |
| **Total biochemicals 0.05≤*p*≤0.10** | 17 | 23 | | 12 | | 25 | | 38 | | 27 | | 28 | | 35 |
| **Upregulated biochemicals** | 15 | 21 | | 7 | | 20 | | 37 | | 12 | | 14 | | 22 |
| **Downregulated biochemicals** | 2 | 2 | | 5 | | 5 | | 1 | | 15 | | 14 | | 13 |
| **Welch’s Two-Sample *t*-Test** | **Wnt1 vs. PyMT** | | **Wnt1 vs/ PyMT-DB** | | **Wnt1 vs. Her2/neu** | | **Wnt1 vs. C3-TAg** | | **PyMT-DB vs. PyMT** | | **C3-TAg vs. PyMT** | | **C3-TAg vs. PyMT-DB** | |
| **Total biochemical *p*≤0.05** | 121 | | 141 | | 122 | | 88 | | 45 | | 93 | | 83 | |
| **Upregulated biochemicals** | 55 | | 66 | | 47 | | 74 | | 28 | | 21 | | 15 | |
| **Downregulated biochemicals** | 66 | | 75 | | 75 | | 14 | | 17 | | 72 | | 68 | |
| **Total biochemicals 0.05≤*p*≤0.10** | 36 | | 47 | | 27 | | 24 | | 28 | | 30 | | 36 | |
| **Upregulated biochemicals** | 17 | | 20 | | 19 | | 17 | | 21 | | 10 | | 5 | |
| **Downregulated biochemicals** | 19 | | 27 | | 8 | | 7 | | 7 | | 20 | | 31 | |
